# Effects of dietary star anise (*Illicium verum*) supplementation during late gestation and lactation on the performance of multiparous sows and their progeny until 21 days post-weaning

**DOI:** 10.5713/ab.24.0766

**Published:** 2025-02-27

**Authors:** Jeong Hyun Moon, Jae Cheol Jang, Minsoo Park, Yoo Yong Kim

**Affiliations:** 1 Department of Agricultural Biotechnology and Research Institute of Agriculture and Life Sciences, Seoul National University, Seoul, Korea; 2 Division of Animal Science and Institute of Agricultural and Life Science, Gyeongsang National University, Jinju, Korea

**Keywords:** Antioxidant, Imprinting, Piglet, Sow, Star Anise

## Abstract

**Objective:**

This study aimed to investigate the effects of star anise (SA) supplementation during late gestation and lactation on the performance of multiparous sows and their progeny until 21 days post-weaning.

**Methods:**

A total of forty pregnant sows were individually kept in stalls and allocated into two treatment groups using a completely randomized design based on body weight, backfat thickness, and parity. The treatments consisted of either 0% or 0.1% SA supplementation during late gestation and lactation. Following the lactation period, 160 weaned piglets were used to investigate the imprinting effects of SA. The dietary treatment was continued in a manner that the piglets received the same diet as their littermates. Data collected included serum antioxidant status in sows, milk composition, and stress indicators in piglets, measured at various stages.

**Results:**

Sows fed with SA during late gestation exhibited significantly higher serum total antioxidant status (p<0.03). Additionally, SA supplementation increased lactose content in the milk (p<0.01). Piglets from sows fed the SA diet during late gestation showed lower serum cortisol and epinephrine concentrations at weaning (p<0.01, p<0.04, respectively). SA supplementation during lactation further reduced serum cortisol levels in weaning piglets at one day after weaning (p<0.05).

**Conclusion:**

The current experiment indicated that supplementing dietary SA in gestation and lactation diet showed higher serum antioxidant properties in sows and improved the quality of sow milk, consequently enhancing litter performance.

## INTRODUCTION

Weaning is the most critical event in the pork production cycle due to adaptation and stress in response to the simultaneous stressors imposed on piglets [1]. Stressors encountered during weaning include abrupt changes in diet and environment, mixing of non-littermates, lack of appropriate stimuli, separation from sow, as well as weaning at an age much earlier than natural [2].

Given these challenges, alternative strategies to mitigate weaning stress have become essential, especially as the use of antibiotics as growth promoters has been largely prohibited [3]. One such strategy is flavor imprinting in young animals’ diets. Numerous studies have shown that prenatal exposure to certain flavors, derived from the maternal diet, can modulate food preferences and neophobias in young animals across various species [4–5]. In these species, the introduction of flavor into the amnion either by direct infusion or maternal ingestion affects later infantile responses to the same stimuli [6]. Star anise (SA; *Illicium verum*), an herbaceous annual plant, contains essential oils, primarily anethole, which constitutes 80% to 90% of its volatile oil [7]. In swine, several studies indicated that anise flavor exposure in lactating sows enhanced insulin-like growth factor-1 (IGF-1) concentration in the sow milk, increased prolactin in the serum of sows, and resulted in higher milk yield [8]. For piglets, it reduced stress-related behaviors [9], increased post-weaning feed intake [10], and led to higher average daily gain (ADG) [8].

Despite the extensive research on the effects of dietary SA supplementation in sow diets during lactation, much of the focus has been on its immediate impact on the growth performance of litters, with less attention given to its sequential effects during the post-weaning period. This study hypothesizes that incorporating SA into sow diets during late gestation and lactation could enhance feed intake and antioxidant status, as well as provide flavor continuity through the piglets’ lactation phase. Such continuity is expected to positively influence the mitigation of weaning stress and improve growth performance in the post-weaning period.

## MATERIALS AND METHODS

All experimental procedure performed in this study was approved by the Animal Care and Use Committee of the Seoul National University (SNU-200419-1). Two trials were conducted with pigs in a multisite production system. The sow experiment was conducted at the Jacob Swine Research Farm, located in Eumseong-gun, Chungcheongbuk-do, Korea. The weaning pig experiment was conducted at the Dae-woo Swine Research Farm, located in Muan-gun, Jeollanam-do, Korea. The distance between two locations is about 320.6 km and takes 4 hours to drive.

This study utilized a plant extract known as RevestArom 1033 Anise P (BIOMIN Phytogenics GmbH, Stadtoldendorf, Germany), which comprises 15.0% natural flavor extracts from SA (*Illicium verum*). The remaining 85.0% of the product consists of binder and carrier materials, specifically silica dioxide and sodium chloride.

### Procedure

**Figure 1:**
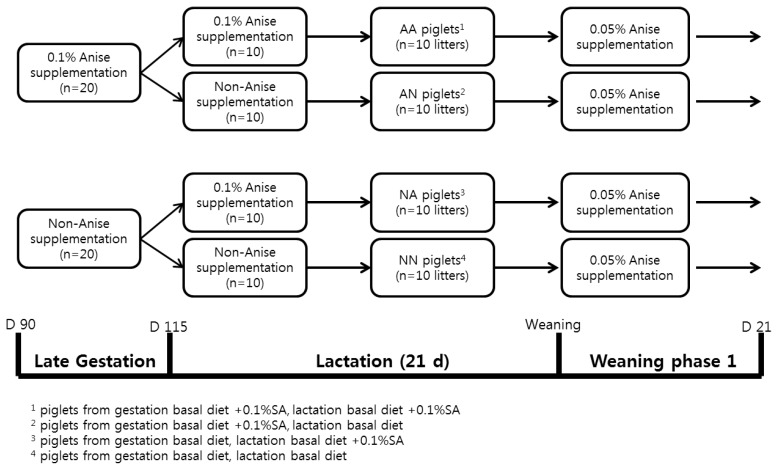
The experimental design and timeline of feeding regimen.

A total of 40 multiparous gestating sows (Yorkshire×Landrace) were used in a 2×2 factorial design experiment. The factors were supplementation of SA either in late gestation and lactation period, respectively. An overview of all experimental treatments and procedures is given in Figure 1. Experimental sows were introduced into individual gestation stalls in an environmentally controlled barn. Estrus was diagnosed twice daily in the presence of a mature boar, using the backfat pressure test. Sows were twice served artificial insemination (AI) with fresh diluted semen (Darby AI center, Chungju-si, Chungcheongbuk-do, Korea) at 12-hour intervals. Pregnancy of the gilts was diagnosed by an ultrasound analyzer (Easyscan; Dong-jin BLS Co., Ltd., Gwangju, Korea) on d 28 and 35 postcoitum.

On day 90 of gestation, the sows were allocated based on body weight (BW), backfat thickness (BFT) and parity in a completely randomized design to one of two treatments, either 0% or 0.1% SA. Sows assigned SA treatments received supplemental SA in their feed drop boxes as a top-dress. On day 110 of gestation, sows were introduced into individual farrowing crates (2.2×1.5 m). Also, daily feed intake was gradually reduced by 0.5 kg/day for each sow until the day of delivery.

Within 24 h post-farrowing, sows in each treatment group were assigned randomly to corn-and soybean meal-based lactation diets supplemented with either 0% or 0.1% SA. The lactation diet was gradually increased from 1.0 kg/d by 0.5 kg/d until 5 d postpartum with a free access to water. For piglets, procedures including Fe-dextran (150 ppm) injection, ear notching, needle teeth clipping, and tail docking were practiced. Additionally, piglets were cross-fostered across treatments within 3 d after birth to balance suckling intensity across sows with equalization of litter size, and thus to minimize any impact of initial litter size potentially affecting litter growth.

On the 21 d after birth, piglets were weaned and subsequently relocated to a different facility. Specifically, a total of 10 litters were chosen from each of the 4 sows participating in the four dietary regimens, totaling 160 piglets ([Yorkshire× Landrace]×Duroc, weighing 7.37±0.98 kg). This selection was conducted to examine the successive imprinting impact of SA derived from the sows’ diets on both the growth performance and the antioxidant status of their offspring. All weaning pigs were top-dressed same amount of SA (0.05%) in the corn-soybean meal-based diet until d 21 post-weaning (Figure 1).

### Housing

The experimental sows were housed in a gestation barn with an individual crate (2.15×0.6 m) with a fully slatted concrete floor. Air temperatures and ventilation rates were measured and determined with sensors, which were installed near the sows and were manipulated by an automatic climate control system (KO-850; KUNOK Co., Ltd., Nonsan, Korea). The average temperature during the entire experimental period was 20.0°C. Feed was accurately weighed by a scale (SW-1W; CAS Co. Ltd., Yangju, Korea), and was provided twice a day (08:00 and 16:00) by feed buckets through an individual feeder with one waterer per sow.

At d 110 of gestation, all sows were moved to the farrowing crates (2.20×0.65 m) with partition walls (2.50×1.80 m) after washing and disinfecting their body. During lactation, the room temperature and air conditioning of the farrowing barn were kept automatically at 25±3°C by heating lamps and ventilation fans. After weaning, sows were moved to the breeding barn again for the next conception.

After weaning, pigs were housed in a 1.2×3.6 m plastic floor, equipped with a feeder and a nipple drinker to allow freely access to feed and water during the three-week experimental period. The ambient temperature in the weaning house was kept 32°C during the first 7 days and lowered 1°C every week.

### Experimental diets

The ingredient composition and calculated nutrient content of diets are shown in Table 1. An additional 0.1% of SA was top-dressed on both gestation and lactation control diet. Gestation diet contains 3,075 metabolizable energy (ME) kcal/kg and 13.0% of crude protein (CP), 0.79% of lysine, 0.49% of methionine, 0.57% of threonine, and 0.15% of tryptophan, respectively. Lactation diet contains 3,116 ME kcal/kg and 17.7% of CP, 1.02% of lysine, 0.63% of methionine, 0.66% of threonine, and 0.22% of tryptophan, respectively. Gestation diets were provided daily at 2.4 kg/day, but lactation and weaner diet were provided *ad libitum*. Weaning pigs’ diets contains 3,382 ME kcal/kg and 19.89% of CP, 1.56% of lysine, 0.89% of methionine, 0.96% of threonine, and 0.26% of tryptophan, respectively. 0.05% of SA was equally top-dressed for all treatment (Table 2). All other nutrients were met or exceeded requirements of NRC [11].

### Measurements and sample collection

In the gestation barn, BW and BFT of sows were measured at days 90, and 110 of gestation and 12 h, 21 d postpartum. BFT was measured at the P2 position (last rib, 65 mm from the center line of the back) on both sides of the back bone using an electric measuring device (Lean-Meater; Renco Corp., Minneapolis, MN, USA). Values from the two measurements were averaged to record a single BFT. Litter traits included the number of piglets born alive, stillborn, mummies, and losses. Within 24 h after birth, the litters were weighed individually by scale (SW-1W; CAS Co. Ltd., Yangju, Korea). Litter and mean pig birth BW, weaning BW, and mean BW gain from birth-to-weaning were calculated. To observe piglet uniformity, coefficient of variation, and standard deviation was calculated at piglet birth and weaning BW. All sows were moved to gestation stalls as soon as all piglets were weaned (approximately 21 days). BW and feed consumption of weaning pigs were recorded at d 3, 7, 14 and 21 post-weaning to calculate ADG, average daily feed intake (ADFI) and gain to feed ratio (G:F ratio).

Sow blood collection was taken by venipuncture of the jugular vein using 10 mL disposable syringes at the same time of measuring the BW and BFT. Piglet (4 piglets per sows, n = 160) blood was collected from the anterior vena cava using 3 mL disposable syringes at 12 h, 21 d postpartum. All samples were enclosed into serum tube (SSTTMII Advance, BD Vacutainer; Becton Dickinson, Plymouth, UK) as well as ethylenediaminetetraacetic acid tubes (BD Vacutainer K2E, Becton Dickinson, Plymouth, UK) and centrifuged at 960×g and 4°C for 5 min after clotting at room temperature for 30 min (5810R; Eppendorf, Hamburg, Germany). The upper liquid (serum) of the blood was separated to a microtube (Axygen, Union City, CA, USA) and stored at −20°C until later analysis.

Sow milk samples were taken from functional mammary glands of each treatment at 24 h after delivery as initial (after cross-fostering), and at 21 d postpartum. Sow milk were collected from the first and second teats after an intravascular injection with 5 IU oxytocin (Komi oxytocin inj.; Komipharm International Co., Ltd., Siheung, Korea) in the ear. After collection, samples were stored in a freezer (−20°C) until further analysis. Proximate analysis of colostrum and milk was determined using a Milkoscan FT 120 (FOSS, Hillerod, Denmark).

### Chemical analysis

The serum superoxide dismutase (SOD) and glutathione peroxidase (GPx) activities in sow serum were measured using commercial assay kit (Cayman Chemical, Ann Arbor, MI, USA) according to the manufacturer’s instructions. Hemoglobin concentration (for estimation of SOD and GPx activities) was measured in a microplate reader (Versamax; Molecular device, San Jose, CA, USA). Total antioxidant status (TAS) was measured using an automated method [12]. Plasma for cortisol concentration was measured using a commercially available ELISA kit (swine cortisol ELISA kit; Endocrine Technologies, Newark, CA, USA). Plasma epinephrine and norepinephrine were assayed using an ion-exchange purification procedure followed by liquid chromatography with electrochemical detection [13]. Briefly, the samples were loaded onto cationic columns, and the catecholamines were eluted with boric acid. The eluates were assayed via HPLC with electrochemical detection with an oxidizing potential of +0.65V.

### Statistical analysis

Performances of SA supplementation in late gestation including physiological response and serum oxidative status were analyzed via pairwise T-test, lactation performance including physiological response, reproductive performance, as well as blood and milk analysis were analyzed via two-way analysis of variance. Individual sow was considered as the experimental unit.

Data of weaning pigs including growth performance and blood analysis were analyzed as a randomized complete block design with two-way ANOVA. The pen of pigs was used as the experimental unit in growth performance, and individual piglet was used as the experimental unit in blood profiles. The significant difference was set at p<0.05, and tendencies were determined if 0.05<p<0.10. All the data was analyzed by the General Linear Model procedure of SAS (version 9.4; SAS Institute Inc., Cary, NC, USA).

## RESULTS

### Physiological response and reproductive performance of gestating sows

In late gestation, we found no significant difference on physiological response in relation to the effect of supplementing SA (Table 3). However, TAS activity at d 110 was significantly higher (p<0.03) in Anise treatment (Table 4). Neither the reproductive performance nor piglet uniformity was affected by SA supplementation (Table 5). Moreover, no gestation× lactation diet interaction was observed.

### Physiological response and reproductive performance of lactating sows

Sows fed SA in late gestation tended to increase BFT within 24 hours of parturition. Likewise, sows consumed SA in both gestation and lactation tended to have much loss in backfat during lactation period (p = 0.10 in gestation, p = 0.06 in lactation, respectively, Table 6). There was a tendency of increased lactation ADFI in the sows fed SA in late gestation (p = 0.08). While sows fed SA during lactation increased number of weaning pigs (p<0.02), sows fed SA during late gestation tended to increase litter weight at 21 d of lactation as well as significantly increased litter weight gain (p = 0.06, p<0.04, respectively).

Table 7 showed the effects of SA supplementation on the serum oxidative status. Sows consumed SA in late gestation tended to have lower TAS activity at 21d lactation (p = 0.09). In piglets at 21 d lactation, a reduced serum GPx activity was observed in both treatments, which sows fed SA both in gestation and lactation (p<0.02, p<0.01, respectively).

Table 8 showed the milk composition of lactating sows fed diets with or without supplemental SA. Compositions of colostrum and ordinary milk were not affected by dietary treatment on casein and fat. On the other hand, sows consumed SA in late gestation tended to have lower protein in sow milk (p = 0.07). In contrast, supplying SA diet during gestation increased lactose in the milk (p<0.01). Solid not fat of the sow milk tended to decreased as SA supplemented in the lactation diet (p = 0.08, respectively). Gestation×lactation effect was noted in the total solid and free fatty acid (FFA) concentration of sow milk at 21d lactation (p = 0.06, p<0.04, respectively).

### Piglet stress level during weaning

Supplying SA diet during late gestation reduced concentration of piglet serum cortisol and epinephrine at weaning (p<0.01, p<0.04, respectively, Table 9). Furthermore, there was a gestation×lactation effect in the piglet serum cortisol concentration (p<0.01).

### Weaning pig growth performance and blood antioxidant level

The effects of SA supplementation during late gestation or lactation diet on the growth performance of weaning pigs were presented in Table 10. There were no significant effects observed on BW, ADG, and ADFI. However, supplementing SA during late gestation tended to enhance G:F ratio of weaning pigs at one to two weeks (p = 0.08). Likewise, supplementing SA during lactation increased G:F ratio in two to three weeks (p<0.05).

The effects of SA supplementation during late gestation or lactation diet on the blood analysis of weaning pigs were summarized in Table 11. The SA supplementation during lactation reduced serum cortisol concentration of weaning pigs in initial phase (p<0.05). Additionally, tendency on gestation× lactation in this stage was observed (p = 0.06). Main effects of gestation, lactation, and gestation×lactation interactions were noted in serum SOD activity at initial and three week of weaning pigs (p<0.01, respectively).

## DISCUSSION

This experiment investigated the effects of supplementing SA in sow diets during late gestation and lactation on physiological responses and reproductive performance, as well as the subsequent growth and health of their progeny during the post-weaning period. Our results indicated that supplementation of SA in the gestation diet increased serum antioxidant status, while supplementation during the lactation diet improved milk quality in sows, leading to an overall enhancement in piglet growth performance. Additionally, postnatal exposure to SA in pigs resulted in decreased stress-related hormones at weaning, which enhanced feed efficiency and improved serum antioxidant status, as indicated by increased SOD levels.

The placenta is known to be a major source of oxidative stress during pregnancy [14]. Oxidative stress occurs when cellular reactive oxygen species (ROS) and free radicals overwhelm the antioxidant defense mechanisms, resulting in macromolecular damage to proteins, lipids, and DNA [15]. This stress increases as gestation progresses, potentially leading to various pregnancy complications such as preterm labor, fetal growth restriction, preeclampsia, and miscarriage [16]. Anethole, an extract from SA, is a member of the phenolic compound family. Although the exact mechanisms behind its effects are not fully understood, several studies have demonstrated a significant linear correlation between total antioxidant capacity and phenolic content, highlighting the effectiveness of phenolic compounds in scavenging hydrogen peroxide, superoxide, and free radicals [17]. Evidence from studies by Wang et al [8] indicated that sows fed 0.5% SA from late gestation through lactation showed enhanced serum antioxidant status Our data corroborate these findings, confirming that SA functions as an antioxidant, promoting the repair and maintenance of damaged cellular membranes and offering protection from oxidative damage during late gestation.

Current experiment indicated that sow ADFI during lactation was positively affected by dietary SA. This finding is consistent with previous reports, which indicated a 4% increase in voluntary feed intake in sows fed a diet supplemented with SA during lactation compared to control diet [8]. Similarly, broilers showed a significant improvement in ADFI with a 0.1% SA-supplemented treatment [18]. These results suggest that the inclusion of SA in sow diets may primarily function as an appetite stimulant, preventing excessive backfat loss during lactation and enhancing the nutritive value of milk provided to piglets.

Litter weight gain and the number of pigs weaned are known to be associated with milk production and nutrient concentration in milk [19]. The observed increase in piglet and litter weight gain in sows supplemented with SA suggests an enhancement in milk production or an increase in nutrient concentrations in the milk. SA has been historically used to increase milk secretion in humans and other animals, classifying it as a galactagogue substance [20]. This property is explained by its pharmacologic effects through interactions with dopamine receptors, which inhibit the secretion of the milk-producing hormone prolactin [21]. Anethole, a component of SA, may influence milk secretion by competing with dopamine at receptor sites, thereby inhibiting dopamine’s anti-secretory action on prolactin [22]. Wang et al [8] reported that SA supplementation during lactation improved piglet weaning weight and milk yield, primarily due to increased concentrations of IGF-1 in sow milk and prolactin in sow serum. Although our study did not include hormonal analyses, it can be inferred that the increased nutrient concentrations, specifically lactose, in sow milk due to SA supplementation contributed to improved litter weight gain and a higher number of weaned pigs. This is consistent with studies by Matysiak et al [23] which showed that a plant extract comprising carvacrol, cinnamaldehyde, and capsicum oleoresin in the lactation sow diet increased milk lactose concentration, potentially preventing hypoglycemia and reducing piglet mortality. One possible mechanism for the increased lactose concentration in sow milk due to SA supplementation can be explained by research from Kreydiyyeh et al [24] which indicated that anise seed oil significantly enhanced glucose absorption in the jejunum by increasing Na+–K+ ATPase activity, which is expected to elevate the sodium gradient. This may stimulate glucose uptake dynamics by the porcine mammary gland for milk production, thereby increasing milk lactose and fatty acid concentrations. Experimental evidence supports that plasma glucose contributes 37% to milk fatty acid synthesis and 60% to 70% to lactose synthesis, largely due to the inverse proportion of insulin concentration during lactation [25].

In this study, we evaluated the activities of serum antioxidant enzymes, including SOD, GPx, and the TAS. These enzymes serve as the primary defense against ROS and are considered reliable indicators of oxidative stress [26]. Variations in these enzymes’ levels, both increases and decreases, have been observed in various diseases due to either an upregulation of enzyme activity or the consumption of these enzymes to neutralize increased ROS levels [15,27]. In our study, supplementing sow diets with SA during late gestation and lactation resulted in enhanced serum antioxidant status and reduced stress-related hormones in piglets. This aligns with previous research indicating that SA can bolster antioxidant enzyme activity, as observed by Ding et al [28], who reported that SA supplementation significantly increased the expression of antioxidant enzymes such as SOD and GPx in poultry. These enzymes are critical in mitigating oxidative stress by neutralizing ROS. Moreover, the enhancement of antioxidant capacity in sows may be attributed to the activation of the Nrf2 signaling pathway, a mechanism that has garnered considerable attention due to its role in regulating cellular antioxidant responses. Ding et al [28] highlighted that dietary SA could upregulate Nrf2 expression, thereby increasing the transcription of genes encoding essential antioxidant enzymes. This mechanism likely underpins the observed improvements in serum TAS in sows and the subsequent benefits for piglets, including better growth performance and reduced weaning stress. These findings suggest that SA supplementation not only supports antioxidant defense in sows but also conveys benefits to their offspring, potentially through maternal nutrient transfer and flavor imprinting. The positive effects on piglet health and development underscore the potential of SA as a dietary supplement to enhance reproductive and postnatal outcomes.

Inclusion of SA into the diet of sows during late gestation was found to decrease serum cortisol and epinephrine levels in piglets at weaning. Additionally, an interaction between gestation and lactation in serum cortisol levels was observed. It is important to note that the blood samples were collected after a stressful protocol involving a 4-hour drive to a multisite research farm followed by 1 hour of pen allocation, conditions that are highly stressful for weaning pigs. The transport to a different site elevates stress levels and exacerbates the physiological, metabolic, and behavioral impact on piglets. Previous research has documented that transportation can lead to increased serum cortisol and creatine phosphokinase levels, which are indicative of stress responses [29]. These changes have been associated with behavioral shifts, fatigue [30], and weight loss regardless of trip duration [31]. The potential beneficial effects of adding SA to the sow’s diet may have indirect anti-stress impacts on weaning piglets. This is supported by the findings from Karimzadeh et al [32] who observed that anise oil supplementation could inhibit the production of dark neurons in the rat brain under stress conditions, such as acute physical stress and aging in the cerebellum. Dark neurons arise from disturbances in the ion gradient (involving the Na/K ATPase pump) and elevated glutamate levels, which can be curtailed by anise oil, thus offering neuroprotective benefits, and potentially reducing weaning stress in piglets [32]. In this experiment, piglets indirectly ingested or were exposed to SA via the diet of their dams, which may have impeded the formation of dark neurons and helped to mitigate weaning stress. Similar phenomena have been observed in other mammals, where neonates recognize flavors not only through inhalation and ingestion of amniotic fluid but also through extracorporeal secretions such as saliva, body odors, feces, or urine [33].

In placental mammals, phenolic compounds like anethole are capable of being transmitted to amniotic fluid or the placental bloodstream, allowing the fetus to develop olfactory and gustatory preferences through a process known as maternal imprinting [9]. Langendijk et al [34] observed that piglets whose mothers had ingested a diet containing garlic and aniseed during both antenatal and postnatal periods showed a marked preference for similarly odorized food over control diets This finding is echoed in research by Oostindjer et al [35] who noted that exposure to anise flavor is more effective when it occurs both before and after birth rather than in the postnatal period alone. Recent studies have also shown that prenatal exposure to specific flavors in the diet can influence feed preferences post-weaning, potentially increasing feed intake and subsequent growth [36].

However, results from our current experiment indicate that piglets exposed to anise postnatally did not show significant differences in feed intake, except for improved feed efficiency in the final phase (weeks 2 to 3). The variation in feed preference attributable to imprinting effects can be explained in several ways. First, due to the shared housing conditions in our experimental setup, there was a potential for cross-contamination among piglets, which could lead to a uniform response across all treatments. Similar issues have been documented in studies involving humans [37] and rodents [38], where sharing the same environment led to cross-contamination of volatile flavors. Secondly, an optimal level of SA inclusion in the diet of all weaned pigs might have mitigated the specific imprinting effects on growth performance. Nevertheless, serum analysis of antioxidant properties in weaned pigs suggests that the familiarity with the flavor, in conjunction with antioxidant enzymes like SOD, can aid in stress reduction.

Antioxidant enzymes play a crucial role in protecting cells from oxidative stress, which is a major factor in gastrointestinal integrity impairment and associated inflammation [39]. According to Windisch et al [40], phytogenic substances can enhance digestive secretions and gut maturation by boosting anti-inflammatory and antioxidant properties, supporting overall health and growth performance in animals.

## CONCLUSION

Supplementation of SA in the diet of sows during late gestation and lactation improved the oxidative status of the sows and promoted growth in piglets. This supplementation was linked to increased litter weight gain, coinciding with higher concentrations of lactose and FFAs in the milk by day 21 of lactation. For piglets, SA not only mitigated weaning stress but also showed potential to enhance feed efficiency. Consequently, SA appears to be a promising feed flavor additive, providing significant antioxidant benefits for the sow diet. However, additional research is required to fully understand the mechanisms behind its stress reduction capabilities.

## Figures and Tables

**Table 1:** The formulas and chemical composition of gestation and lactation diet (as fed basis)

Item^1)^	Gestation	Lactation
Ingredient (%)	100.0	100.0
Corn (yellow)	72.55	63.70
Soybean meal (45% CP)	14.74	27.10
Wheat mill run	6.0	2.8
Animal fat	2.6	2.8
Monodicalcium phosphate	1.8	1.5
Limestone	1.3	1.3
Lysine sulfate (70%)	0.3	0.1
Salt	0.3	0.3
Choline chloride (50%)	0.1	0.1
L-threonine (98%)	0.1	0.0
Vit. Mix.^2)^	0.1	0.1
Min. Mix.^3)^	0.2	0.2
Chemical composition^4)^		
ME (kcal/kg)	3,075	3,116
Crude protein (%)	13.00	17.70
Calcium (%)	0.85	0.83
Phosphorus (%)	0.71	0.67
Lysine (%)	0.79	1.02
Methionine+cysteine (%)	0.49	0.63
Threonine (%)	0.57	0.66
Tryptophan (%)	0.15	0.22

1) Treatments: gestation diets were fed 2.4kg/day in two separate meals; lactation diets were fed *ad libitum* up to weaning at 21days. Star anise diets were supplemented with 0.1% anise extract.

2) Provided per kg of diet: 1. Gestation: vitamin A, 10,800 IU; vitamin D_3_, 2,400IU; vitamin E, 72 IU; vitamin K, 3.6 mg; vitamin B_2_, 7.2 mg; vitamin B_6_, 4.8 mg; vitamin B_12_, 30 μg; pantothenic acid, 24 mg; biotin, 324 μg; niacin, 48 mg; folic acid 3.12 mg; thiamine, 1.56 mg. 2. Lactation: vitamin A, 9,000 IU; vitamin D_3_, 2,000 IU; vitamin E, 60 IU; vitamin K, 3 mg; vitamin B_2_, 6 mg; vitamin B_6_, 4 mg; vitamin B_12_, 25 μg; pantothenic acid, 20 mg; biotin, 270 μg; niacin, 40 mg; folic acid 2.6 mg; thiamine, 1.3 mg.

3) Provided per kg of diet: Fe, 165 mg; Mn, 60 mg; Zn, 99 mg; Cu, 7.5 mg; Se, 450 μg; I, 1 mg.

4) Calculated value.

ME, metabolizable energy.

**Table 2:** The formula and chemical composition of weaning pig diets (as fed basis)

Item	Control diet
Ingredient (%)	100.00
Corn (yellow)	48.72
Soybean meal (45% CP)	15.00
Permeate SBM (54% CP)	5.00
Whey permeate	6.94
Lactose	5.00
Whey	2.00
Wheat mill run	3.00
Plasma protein	4.50
White fishmeal	2.50
Coconut fat	2.00
Vegetable oil	1.50
Monodicalcium phosphate	0.78
Limestone	0.50
Lysine sulfate (70%)	0.76
Salt	0.30
Choline chloride (50%)	0.14
L-threonine (98%)	0.15
Organic acid	0.40
DL-methionine (98%)	0.19
Zinc oxide (90%)	0.31
Tryptophan (10%)	0.10
Yucca powder	0.01
Vit. Mix.^1)^	0.10
Min. Mix.^2)^	0.10
Chemical composition^3)^	
ME (kcal/kg)	3,400.00
Crude protein (%)	19.89
Total calcium (%)	0.85
STTD phosphorus (%)	0.45
SID lysine (%)	1.50
SID methionine+cysteine (%)	0.85
SID threonine (%)	0.90
SID tryptophan (%)	0.25

1) Provided per kg of diet: vitamin A, 10,000 IU; vitamin D_3_, 2,000 IU; vitamin E, 60 IU; vitamin K, 3.5 mg; vitamin B_2_, 8 mg; vitamin B_6_, 2 mg; vitamin B_12_, 35 μg; pantothenic acid, 25 mg; biotin, 100 μg; niacin, 50 mg; folic acid 3.1 mg; thiamine, 1.5 mg.

2) Provided per kg of diet: Fe, 100 mg; Mn, 50 mg; Zn, 50 mg; Cu, 80 mg; Se, 400 μg; I, 1 mg.

3) Calculated values.

ME, metabolizable energy; STTD, standardised total tract digestible; SID, standard ileal digestibility.

**Table 3:** Effect of star anise supplementation on changes in sow body weight and backfat thickness during gestation

Gestation	Treatment		SEM	p-value

	Control	Anise		
Body weight (kg)				
90 d gestation	254.2	256.9	4.95	0.79
110 d gestation	267.5	274.5	5.04	0.49
BW gain (90–110 d)	13.3	17.6	5.90	0.72
Backfat thickness (mm)				
90 d gestation	25.5	25.2	0.88	0.86
110 d gestation	25.1	28.1	0.83	0.06
BF gain (90–110 d)	−0.4	2.9	1.23	0.18

SEM, standard error of the means; BW, body weight; BF, body fat.

**Table 4:** Effect of star anise supplementation on serum antioxidant enzymes of sow during gestation

Gestation	Treatment		SEM	p-value

	Control	Anise		
90 d				
GPx (nmol/min/mL)	416.84	450.48	13.241	0.32
TAS (mmol/L)	0.76	1.18	0.303	0.62
SOD (U/mL)	2.14	2.58	0.627	0.49
110 d				
GPx (nmol/min/mL)	505.99	522.03	32.260	0.82
TAS (mmol/L)	0.90^b^	0.95^a^	0.013	0.03
SOD (U/mL)	3.84	3.40	0.383	0.60

a,b Means with different superscripts in the same row significantly differ.

SEM, standard error of the means; GPx, glutathione peroxidase; TAS, total antioxidant status; SOD, superoxide dismutase.

**Table 5:** Effect of star anise supplementation on reproductive performance of sows

Gestation	Control	Anise	SEM	p-value
No. of piglets				
Total born	13.95	13.01	0.665	0.48
Stillborn	0.86	0.59	0.141	0.29
Mummy	0.17	0.11	0.071	0.62
Born alive	12.93	12.32	0.616	0.65
Uniformity				
Total litter weight (kg)	17.76	17.36	0.747	0.65
No. of piglets/litter	13.25	12.95	0.640	0.68
Average piglet weight (kg)	1.34	1.35	0.038	0.81
Standard deviation	322.90	307.20	16.81	0.52
Coefficient of variation	24.57	23.06	1.393	0.54

SEM, standard error of the means.

**Table 6:** Effect of star anise supplementation on physiological response and reproductive performance of sow during lactation

Item	Gestation		Lactation		SEM	p-values^1)^		
	
	Control	Anise	Control	Anise		G	L	G×L
Body weight (kg)								
24 hrs postpartum	243.6	235.6	254.8	247.7	6.57	0.32	0.51	0.96
21 d lactation	233.8	222.9	244.1	246.4	8.15	0.11	0.68	0.53
BW changes (0–21 d)	−9.8	−12.7	−10.7	−1.3	4.89	0.38	0.58	0.30
Backfat thickness (mm)								
24 hrs postpartum	25.6	26.9	28.0	26.8	0.86	0.07	0.26	0.66
21 d lactation	24.6	23.4	26.0	25.9	0.87	0.28	0.72	0.76
BF changes (0–21 d)	−1.0	−3.6	−2.0	−0.9	1.55	0.53	0.61	0.78
ADFI kg	4.59	4.60	5.24	5.15	0.368	0.41	0.90	0.88
No. of piglets								
After-fostering	11.5	11.6	11.3	11.6	0.14	0.81	0.57	0.74
d-21 lactation	9.6	10.9	10.1	11.2	0.25	0.38	0.02	0.86
Litter weight (kg)								
24 hr postpartum	16.33	15.91	16.68	15.63	0.354	0.96	0.32	0.67
21 d lactation	48.11	56.58	59.18	59.43	1.904	0.06	0.24	0.26
Weight gain (0–21 d)	31.78	40.67	42.50	43.80	1.801	0.04	0.14	0.27
Piglet weight (kg)								
24 hr postpartum	1.43	1.38	1.47	1.35	0.030	0.97	0.17	0.53
21 d lactation	5.08	5.23	5.81	5.35	0.155	0.18	0.63	0.33
Weight gain (0–21 d)	3.65	3.85	4.34	4.00	0.144	0.15	0.81	0.36

1) G, supplementing 0.1% SA during gestation; L, supplementing 0.1% SA during lactation; G×L, interaction between SA supplementation during gestation and lactation.

SEM, standard error of the means; ADFI, average daily feed intake; SA, star anise.

**Table 7:** Effect of star anise supplementation on serum antioxidant enzyme in sows and piglets at d 21 lactation

Item	Gestation		Lactation		SEM	p-values^1)^		
	
	Control	Anise	Control	Anise		G	L	G×L
Sow at 21d lactation								
GPx (nmol/min/mL)	244.4	236.7	303.1	263.1	22.09	0.38	0.62	0.73
TAS (mmol/L)	0.99	0.99	0.96	0.97	0.008	0.09	0.81	0.81
SOD (U/mL)	3.36	3.16	3.59	3.55	0.306	0.65	0.85	0.91
Piglet at 21 d lactation								
GPx (nmol/min/mL)	522.5	429.6	471.4	350.9	19.57	0.02	0.01	0.58
TAS (mmol/L)	0.72	0.76	0.85	0.73	0.035	0.49	0.53	0.28
SOD (U/mL)	4.99	5.02	5.01	4.89	0.151	0.87	0.88	0.83

1) G, supplementing 0.1% SA during gestation; L, supplementing 0.1% SA during lactation; G×L, interaction between SA supplementation during gestation and lactation.

SEM, standard error of the means; GPx, glutathione peroxidase; TAS, total antioxidant status; SOD, superoxide dismutase; SA, star anise.

**Table 8:** Effect of star anise supplementation on sow milk composition during lactation

Item (%)	Gestation		Lactation		SEM	p-values^1)^		
	
	Control	Anise	Control	Anise		G	L	G×L
Casein								
Initial (after-fostering)	6.98	6.92	7.01	7.30	0.125	0.21	0.46	0.54
Final (21 d lactation)	4.67	4.40	4.39	4.46	0.056	0.31	0.40	0.14
Fat								
Initial (after-fostering)	6.97	7.21	7.32	6.88	0.328	0.66	0.32	0.25
Final (21 d lactation)	6.19	5.95	5.87	6.98	0.223	0.42	0.33	0.14
Protein								
Initial (after-fostering)	9.10	9.32	9.68	8.97	0.321	0.42	0.36	0.28
Final (21 d lactation)	5.29	4.99	4.87	4.91	0.071	0.07	0.33	0.20
Lactose								
Initial (after-fostering)	4.32	4.52	4.23	4.37	0.147	0.24	0.45	0.37
Final (21 d lactation)	6.24	6.10	6.40	6.32	0.041	0.01	0.12	0.64
Total solid								
Initial (after-fostering)	22.18	22.32	22.75	23.48	0.568	0.66	0.32	0.24
Final (21 d lactation)	19.35	18.45	18.65	19.74	0.260	0.56	0.84	0.06
Solid not fat								
Initial (after-fostering)	13.70	13.45	13.64	13.32	0.184	0.24	0.17	0.20
Final (21 d lactation)	11.50	11.27	11.36	11.17	0.059	0.29	0.08	0.86
Free fatty acid								
Initial (after-fostering)	6.14	5.26	5.27	4.98	0.547	0.17	0.15	0.32
Final (21 d lactation)	8.24	5.14	7.78	8.20	0.488	0.12	0.12	0.04

1) G, supplementing 0.1% SA during gestation; L, supplementing 0.1% SA during lactation; G×L, interaction between SA supplementation during gestation and lactation.

SEM, standard error of the means; SA, star anise.

**Table 9:** Effect of star anise supplementation in sow diet on serum stress status of piglet at the weaning

Item	Gestation		Lactation		SEM	p-values^1)^		
	
	Control	Anise	Control	Anise		G	L	G×L
Cortisol (μg/dL)	6.32	3.80	2.62	3.80	0.463	0.01	0.34	0.01
Epinephrine (pg/mL)	68.15	43.80	26.67	25.75	7.402	0.04	0.35	0.39
Norepinephrine (pg/mL)	40.12	23.87	32.25	34.47	4.091	0.87	0.42	0.29

1) G, supplementing 0.1% SA during gestation; L, supplementing 0.1% SA during lactation; G×L, interaction between SA supplementation during gestation and lactation.

SEM, standard error of the means; SA, star anise.

**Table 10:** Effect of star anise supplementation in sow diet and weaning diet on piglet performance

Item	Gestation		Lactation		SEM	p-values^1)^		
	
	Control	Anise	Control	Anise		G	L	G×L
Body weight (kg)								
Initial	7.36	7.36	7.37	7.37	0.214	0.92	0.44	0.38
1 week	8.98	8.51	8.63	8.54	0.700	0.81	0.67	0.77
2 week	12.15	11.05	11.66	11.38	0.361	0.92	0.44	0.64
3 week	16.01	14.96	15.73	15.20	0.420	0.98	0.45	0.80
Average daily gain (g)								
0–1 week	231	164	180	167	13.5	0.45	0.22	0.40
1–2 week	454	362	433	405	16.3	0.77	0.13	0.40
2–3 week	552	559	581	547	11.8	0.77	0.64	0.49
Overall	412	362	398	373	11.3	0.95	0.18	0.65
Average daily feed intake (g)								
0–1 week	226	192	191	187	8.5	0.27	0.31	0.42
1–2 week	516	464	486	459	16.9	0.65	0.31	0.75
2–3 week	816	722	808	733	23.5	0.97	0.13	0.85
Overall	519	459	495	459	14.3	0.70	0.14	0.70
Gain-to-feed ratio								
0–1 week	0.972	0.846	0.904	0.881	0.0417	0.71	0.37	0.49
1–2 week	0.875	0.778	0.893	0.885	0.1452	0.08	0.13	0.19
2–3 week	0.679	0.786	0.724	0.744	0.0141	0.95	0.05	0.16
Overall	0.794	0.791	0.803	0.808	0.0103	0.59	0.96	0.86

1) G, supplementing 0.1% SA during gestation; L, supplementing 0.1% SA during lactation; G×L, interaction between SA supplementation during gestation and lactation.

SEM, standard error of the means; SA, star anise.

**Table 11:** Effect of star anise supplementation in sow and weaning diet on blood profiles of weaning pigs

Item	Gestation		Lactation		SEM	p-values^1)^		
	
	Control	Anise	Control	Anise		G	L	G×L
Cortisol (μg/dL)								
Initial	11.52	6.52	7.61	7.42	0.666	0.23	0.05	0.06
1 week	11.34	9.45	14.71	6.25	1.937	0.98	0.31	0.51
2 week	5.83	6.82	4.85	7.63	0.538	0.94	0.15	0.49
3 week	6.67	7.13	4.67	5.98	0.511	0.14	0.40	0.68
SOD (U/mL)								
Initial	6.90^b^	6.07^c^	6.31^bc^	10.74^a^	0.422	0.01	0.01	0.01
1 week	8.00	6.38	5.29	7.54	0.449	0.45	0.76	0.07
2 week	8.13	7.88	8.79	6.75	0.336	0.75	0.14	0.25
3 week	7.27^c^	9.42^b^	7.56^c^	13.72^a^	0.553	0.01	0.01	0.01

1) G: supplementing 0.1% SA during gestation, L: supplementing 0.1% SA during lactation, G×L: interaction between SA supplementation during gestation and lactation.

a–c Means in a row with different letters differ (p<0.05).

SEM, standard error of the means; SOD, superoxide dismutase; SA, star anise.
